# Serum Metabolites Characterization Produced by Cats CKD Affected, at the 1 and 2 Stages, before and after Renal Diet

**DOI:** 10.3390/metabo13010043

**Published:** 2022-12-27

**Authors:** Bruna Ruberti, Daniela Pedrosa Machado, Thiago Henrique Annibale Vendramini, Vivian Pedrinelli, Pedro Henrique Marchi, Juliana Toloi Jeremias, Cristiana Fonseca Ferreira Pontieri, Marcia Mery Kogika, Marcio Antonio Brunetto

**Affiliations:** 1 Pet Nutrology Research Center, Animal Nutrition and Production Department, School of Veterinary Medicine and Animal Science, University of São Paulo, 225, Duque de Caxias Norte Ave, Pirassununga, São Paulo 13635-900, Brazil; 2 Nutritional Development Center, Grandfood Industria e Comercio LTDA (PremieRpet^®^), Luiz Augusto de Oliveira Hwy, km 204, Dourado, São Paulo 13590-000, Brazil

**Keywords:** chronic kidney disease, felines, metabolic profile, nutrition, renal disease, uremic toxins

## Abstract

Utilizing metabolomics, a tool for measuring and characterizing low-molecular-weight substances (LMWs), to identify eventual changes in response to dietary intervention is novel in cats with chronic kidney disease (CKD), a condition characterized by retention of uremic solutes. This study aims to assess the serum metabolomic profile of cats in early stages of CKD and to compare the serum metabolomic of CKD cats after 60 days of a renal diet to evaluate the effect of dietary intervention on these metabolites. Twenty-five domestic cats were included in the study. Fifteen cats with CKD stages 1 (*n* = 6) and 2 (*n* = 9) according to the International Renal Interest Society (IRIS) were included in the renal groups, and a control group consisting of 10 cats was included. All animals were enrolled on a maintenance diet for 30 days before the experimental period. The metabolomics analysis was performed by gas chromatography-mass spectrometry (GC-MS). Partial least squares discriminant analysis (PLS-DA) was performed on Metaboanalyst 4.0 software. Forty-three metabolites were identified. Citric acid and monostearin were altered in the CKD2 group when compared to CKD1 and the control group at T0. A total of seven serum metabolites differed after 60 days of the renal diet: glycine, fructose, glutamic acid, arachidonic acid, stearic acid, creatinine, and urea. Changes were seen in the serum metabolomic profile after 60 days of the renal diet, and some of the metabolites that changed in response to the diet have beneficial effects on health. Overall, metabolomics markers have the potential to identify early stages of CKD, providing insights into the possible pathophysiologic processes that contribute to the development and progression of CKD.

## 1. Introduction

Chronic kidney disease (CKD) is a highly prevalent ailment in domestic cats, and is characterized by progressive loss of renal function that is associated with high mortality and morbidity [1,2]. The etiology of CKD is heterogeneous, and usually the primary causes are not identified due to the adaptive course of the disease’s evolution [3,4]. Furthermore, the time course of CKD is variable in cats [5]. The clinical signs may be non-specific and only observed in the most advanced stages, after at least 66% of nephrons have been injured or have fully lost their functions [6,7]. Polyuria and polydipsia, dehydration, hyporexia or anorexia, gastrointestinal signs, anemia, lethargy, and cachexia are some of the clinical signs that occur because of the failure to excrete waste metabolites that lead to uremic syndrome [3,8,9]. 

New strategies for early diagnosis of CKD are emerging in veterinary medicine which can aid therapeutic interventions to decrease disease progression. One of the major challenges is the early identification of structural and functional disorders when the animal presents no clinical signs. The metabolomics approach is the study that involves the identification and quantification of small molecules present in biological samples. It aims to show changes in individual metabolism under different conditions, as well as to elucidate the pathophysiological mechanism of chronic diseases such as CKD [10,11].

The idea of utilizing metabolomics to identify molecular signatures, and eventual changes in response to dietary intervention, is novel in cats with chronic kidney disease. The use of therapeutic diets in CKD management is one of the recommendations to control the disease’s progression attenuating the oxidative stress [12,13,14,15] and the inflammatory course of the disease [15,16]. Therapeutical renal diets have reduced protein, phosphorus and sodium content, are enriched with ω-3 polyunsaturated fatty acids (PUFAs), antioxidants, potassium, vitamin B, antioxidants, and soluble fiber and the caloric density is increased [3,17,18,19]. These nutritional characteristics are designed to reduce clinical signs of uremia and improve the survival rate of cats with CKD [20,21,22]. 

This study aimed to assess the serum metabolomics of healthy cats and cats with CKD stages 1 and 2. The serum metabolomics of CKD cats was compared after a period of 60 days of the renal diet to evaluate the effect of dietary intervention on these metabolites.

## 2. Materials and Methods

### 2.1. Animals and Study Design

This study was conducted at the PremieRpet^®^ Nutritional Development Center (Dourado, São Paulo, Brazil) and at the Pet Nutrology Research Center (CEPEN pet) of the School of Veterinary Medicine and Animal Science of the University of São Paulo (FMVZ/USP) (Pirassununga, São Paulo, Brazil). The study was approved by the Ethics Committee of the Veterinary Medicine and Animal Science School of the University of São Paulo (FMVZ/USP), protocol number 5433030719, and the PremieRpet^®^ Nutritional Development Center, protocol number 088-18.

Twenty-five domestic cats were included in the study. Fifteen cats with CKD stage 1 (*n* = 6, 5 males and 1 female) and 2 (*n* = 9, 4 males and 5 females) according to the International Renal Interest Society (IRIS) [9] were included in the renal groups. The control group was composed of 10 healthy cats (*n* = 10, 6 males and 4 females). 

The first experimental group (CKD1) was composed of six cats of various breeds (Exotic, British Shorthair, Bengal, Persian, Abyssinian and half-breed cats), with a mean age of 10.83 ± 1.05 years, mean body weight of 5.29 ± 0.73 kg, mean body condition score (BCS) of 6.15 ± 0.47 [23], and mean muscle mass score (MMS) of 3.00 ± 0.16 [24]. These animals were diagnosed with CKD stage 1 based on the presence of persistent alteration of renal morphology on ultrasound findings, and normal blood levels of creatinine and symmetric dimethylarginine (SDMA) (creatinine < 1.6 mg/dL and SDMA < 18 μg/dL) according to IRIS [9].

The second group was the CKD stage 2 (CKD2) and consisted of nine half-breed cats, with a mean age of 10.22 ± 1.35 years, mean body weight of 4.72 ± 1.51 kg, mean BCS of 5.75 ± 0.42 [23], and mean MMS of 2.30 ± 0.14 [24]. These animals were diagnosed with CKD stage 2 based on persistent azotemia over at least three months, with creatinine ranging from 1.6 to 2.8 mg/dL and/or SDMA levels of 18 to 25 μg/dL according to IRIS [9].

The third group was the control group (CG) and consisted of 10 healthy cats of various breeds (Ragdoll, Bengal, Maine Coon and half-breed cats) with a mean age of 5.30 ± 1.07 years, mean body weight of 4.52 ± 0.65 kg, mean BCS of 5.59 ± 0.37 [23], and mean MMS of 2.84 ± 0.13 [24]. Healthy cats were included in the study based on normal findings from history, physical examination, complete blood count (CBC), serum biochemical profile, urinalysis, and abdominal ultrasound.

### 2.2. Diet and Feeding Protocol

**Figure 1:**
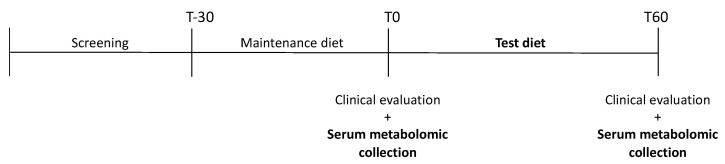
Visual scheme of the experimental schedule.

All observed groups (CKD and control groups) were enrolled on a complete and balanced dry food designed for senior cats (Senior diet; PremieR Gatos Castrados Acima dos 12 anos. PremieR pet, Brazil) for 30 days before the samples were first collected. Subsequently, every group received a Test diet formulated for cats with CKD for 60 days (Renal test diet). To adapt the animals to the new experimental diet, there was a 5-day period when the previous food and the experimental diet were mixed. Sample collections were conducted in two periods, T0 and T60: T0 being collected immediately after 30 days of the senior diet and T60, 60 days after T0 (Figure 1). Both were extruded at PremieRpet^®^ Factory Unit (Dourado, São Paulo, Brazil). The analyzed chemical composition of the diets, expressed in g/100 kcal, is presented in Table 1. 

The diet’s metabolizable energy (ME) content was estimated from the expected chemical composition, and the maintenance energy requirements (MER) were estimated according to the equation MER = 75 × BW^0.67^. Diets were prescribed on gram unit, and the amount of food for each animal was calculated using MER and the diet’s ME. Fresh water was offered *ad libitum*. Cats were housed individually and had opportunities to access an outdoor area to exercise and to access toys, and they interacted with staff from the research centers.

### 2.3. Sample Collection and Preparation

All blood samples were collected by venipuncture in the morning period after 8 h of fasting, and were transferred into red top vacutainer tubes containing a clot activator. 

For gas chromatography-mass spectrometry (GC-MS) analysis, serum was separated by centrifugation (3000 rpm for 10 min) within 30 min of collection and frozen immediately at −80 °C for further analysis at the Metabolomics Laboratory at the Federal University of São Paulo (UNIFESP), São Paulo, São Paulo, Brazil.

### 2.4. GC-MS, Acquisition, Processing Parameters, and Identification of Serum Metabolites

For GC-MS analysis, 100 µL aliquots of samples from each group were vortexed with 300 µL of acetonitrile at 4 °C for deproteinization to occur and centrifuged at high speed. 100 µL of the supernatant was transferred to GC-MS vials containing glass inserts for the derivatization process. For the methoximation step, the solvent evaporated at 30 °C in a SpeedVac and the O-methoxyamine hydrochloride (15 mg/mL) in pyridine added to the vials, vortexed and incubated for 16 h in the dark at room temperature. After this period, silylation started with 10 µL of BSTFA [1% TMCS (*v*/*v*)] and samples were incubated at 70 °C for 1 h. Finally, 20 µL of pentadecanoic acid (20 ppm in heptane) were added to each analysis vial. Blanks were prepared and analyzed to correct the baseline of the chromatograms. These blanks were analyzed at the beginning, middle, and end of the sequence [25,26].

The analyses were carried out in a quadrupole-type GCMS-QP2020NX system (Shimadzu Co., Kyoto, Japan), with 1 µL of the sample loaded into a DB5-MS column (30 m × 0.25 mm, 0.25 μm, Restek) and injected in splitless mode in a total flow of 20 mL/min of helium gas. Carrier gas was conducted at a constant flow of 1.36 mL/min. The initial column temperature was initially maintained at 80 °C and then gradually increased at a rate of 15 °C/min, until reaching the final temperature of 300 °C and then maintained at this temperature for 8 min before cooling. The temperatures of the injector, transfer line and source filament and the quadrupole were maintained at 280 °C, 200 °C, and 150 °C, respectively. The system was operated in full scan mode (*m*/*z* 40–650) at a rate of three spectra/s, and with the EI set to 70 eV. Then, a closed retention time (TRF) method was applied to reduce the retention time (TR) of the entire analysis [25,27]. Instrument control, data acquisition and data processing were performed by LabSolutions software (GCMS version 4.5, Shimadzu Co., Kyoto, Japan), which allows the real-time control of each analyzed analyte for the identification of metabolites in SIM and Scan.

The identification of metabolites was performed by comparing the spectra obtained through the analyzed samples with reference spectra acquired under the same experimental conditions. That is, the peaks of the analytes and external standards were integrated by the same software, using the same settings and normalized by the addition and detection of internal standards added to each sample prior to analysis. Thus, an analytical calibration curve was constructed to determine the linearity, and with the use of internal standards, the concentration for each metabolite generated quantification values in micro molar (uM) with greater reliability. The data were finally exported to Excel software (Microsoft Office) for subsequent statistical analysis of the molecules identified and quantified in SIM mode.

For the analysis in Scan mode, the detected metabolites were processed to create a unified matrix of variables from the different states of charge, adducts and groups of the same analytes across all samples using the GCMS Solution software (v.3.30), NIST 17 MASS (v.1.00.1) and GCMS Smart Metabolite (v.3.01), all developed by Shimadzu Co. The software was configured as efficiently as possible to process all detected peaks, separating them from the equipment noise. After identifying the molecules by the NIST [26] and Smart Metabolite libraries, the samples were exported to Excel software (Microsoft Office) for statistical treatment. If necessary, public databases available on the internet [(www.metlin.scripps.edu, https://www.genome.jp/kegg/, www.lipidmaps.org or http://www.hmdb.ca (accessed on 12 January 2022)] can also be used for the identification and/or conformation of GC-MS spectra.

### 2.5. Statistical Analysis

Data analysis was performed on Metaboanalyst 4.0 software [http://www.metaboanalyst.ca/ (accessed on 23 January 2022)] following parametric and non-parametric algorithms such as T-test, parametric ANOVA and its non-parametric version (Kruskal-Wallis) creating multivariate models with logarithmic transformation for data normalization. Supervised models such as partial least squares regression (PLS) were used.

The Interactome analysis of metabolic pathways of the molecules that were analyzed and considered significant for the proposed experimental model were conducted in the Cytoscape software ware [(https://cytoscape.org/ (accessed on 25 January 2022)]. The level of significance was set to *p*-value ≤ 0.05.

## 3. Results

All cats had a stable renal function, without symptoms such as anorexia or lack of appetite, nausea/vomiting or associated conditions, and underwent a complete physical examination, complete blood count, and biochemical serum profile [total protein, albumin, glucose, creatinine, blood urea nitrogen (BUN), total phosphorus (P), total calcium (Ca), sodium (Na), potassium (K), chloride (Cl), cholesterol, triglycerides, alkaline phosphatase (ALP), and alanine aminotransferase (ALT), urinalysis, and abdominal ultrasound]. A descriptive analysis of the biochemical serum profile is presented in Table 2.

A total of 43 metabolites of different biochemical classes were detected at baseline from both healthy and CKD cats (Table 3). These metabolites were separated according to their classification, identification code, identification level, identification key in the Human Metabolome Database and relative standard deviation (RSD). They were calculated from Quality Control samples (QCs), prepared and analyzed with the animal samples to validate the repeatability and robustness of the method, the injection precision, and the analytical variation of the samples. Metabolites found with a relative standard deviation above 30.9% were removed from the analysis.

### 3.1. Partial Least Squares (PLS) for Serum Metabolomics Data at Baseline (T0) and after Sixty Days of Renal Diet (T60) between CKD Cats (Stages 1 and 2) and Control Group

**Figure 2:**
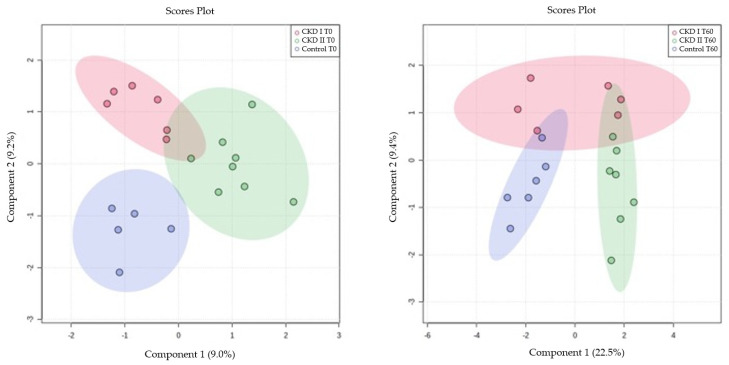
Partial Least Squares (PLS) for serum metabolomics data at baseline (T0) and after sixty days of renal diet (T60). CKD I T0 = CKD cats stage 1 before being fed a renal diet; CKD II T0 = CKD cats stage 2 before being fed a renal diet; Control T0 = control group (healthy animals) before being fed a renal diet; CKD I T60 = CKD cats stage 1 after being fed a renal diet; CKD II T60 = CKD cats stage 2 after being fed a renal diet; Control T60 = control group (healthy animals) after being fed a renal diet.

A multivariate analysis of the CKD1, CKD2, and control groups was performed at T0 and T60 using the Partial Least Squares (PLS), which demonstrated the disposition of the animals classified according to the metabolomics of each animal (Figure 2).

### 3.2. Univariate Analysis for Serum Metabolomics Data at Baseline (T0) and after Sixty Days of Renal Diet (T60) between CKD Cats (Stages 1 and 2) and Control Group

**Figure 3:**
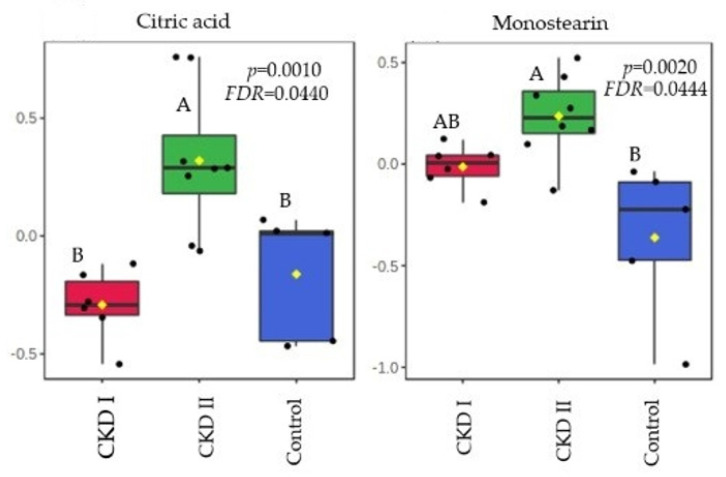
Metabolites that significantly differed among groups before of consumption of the renal diet. CKD I = CKD cats stage 1; CKD II = CKD cats stage 2; Control = control group (healthy animals).

**Figure 4:**
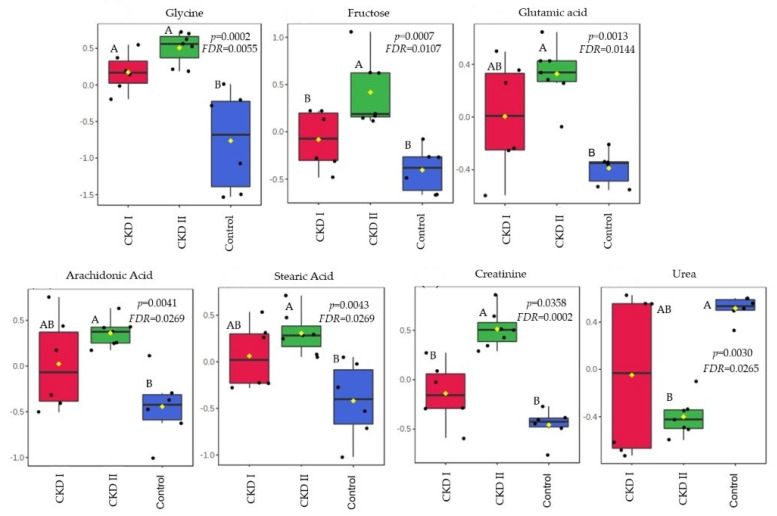
Metabolites that significantly differed among groups after 60 days of consumption of the renal diet. CKD I = CKD cats stage 1; CKD II = CKD cats stage 2; Control = control group (healthy animals).

Citric acid and monostearin were the metabolites that were altered in the CKD2 group when compared to CKD1 and the control group in T0. A total of 7 serum metabolites differed after consumption of the renal diet compared with the maintenance diet (T60): glycine, fructose, glutamic acid, arachidonic acid, stearic acid, creatinine, and urea (Table 4). Figure 3 and Figure 4 show which groups differed between the two periods.

## 4. Discussion

Using an untargeted metabolomics approach, a total of 43 metabolites of different biochemical classes were detected in serum at baseline from both healthy cats and cats with CKD. Citric acid and monostearin were the metabolites that differed in T0.

Citric acid is an intermediate metabolite from the tricarboxylic acid (TCA) cycle being synthesized from acetyl-CoA and oxaloacetate. These metabolic pathways occur in the mitochondrial matrix, which is responsible for cellular energy production due to the adenosine triphosphate (ATP) generation [28]. Kidney cells are rich in mitochondria and are highly dependent on this organelle’s function due to their high energy demand [28,29].

In humans, some studies revealed that the severity and progression of CKD is associated with mitochondrial dysfunction and markers of oxidative stress [29,30]. If mitochondrial activity is unbalanced, there is an increase in oxidative stress and the inflammatory state, which may be more pronounced in CKD2 patients [28,29,30]. Hence, mitochondrial and TCA cycle alterations can be associated with an accumulation of serum citric acid, which is a precursor of several other metabolites [31].

In addition, the stimulation of another pathway to compensate the lack of ATP from glucose sources may occur [32]. An adaptation of the metabolism in relation to the energy source (shifting from glucose to lipids) may be responsible for the increase in monostearin, which is a fatty acid (monoglyceride) and was the second metabolite that increased in CKD2 compared to healthy patients [32,33]. This information corroborates with a study performed by Wei et al. [34], who observed changes in metabolic profiles during periods of renal ischemia. The kidney and plasma showed evidence of altered energy metabolism affecting glycolysis, the TCA cycle and lipid metabolism, and there was a notable switch of energy source from glucose to lipids [34]. Huang et al. [32] also demonstrated through an integrated proteomic and metabolomic approach, that there is an activation of another pathway of energy source in cases of kidney injury to compensate for the function of injured kidneys. In both studies, monostearin showed an increase in concentration which corroborates our findings [32,34].

Another possibility for this result is that the fatty acids oxidation is decreased in CKD patients. Lipidic disorders are common in human patients with kidney disease. A study conducted by Vaziri [35] demonstrated that during the progression of CKD in humans, it is common that the patients present hypercholesterolemia and elevated low-density lipoprotein (LDL) levels. In veterinary medicine, dyslipidemia has already been reported in CKD dogs [36]. However, the cats from our study did not show any difference between groups in relation to the concentrations of cholesterol and triglycerides.

A total of 7 serum metabolites differed after 60 days of the renal diet compared with the maintenance diet: glycine, fructose, glutamic acid, arachidonic acid, stearic acid, creatinine, and urea. All of them were increased in the CKD stage 2 group, except for urea which was lower in the CKD2 when compared to the other groups.

The kidneys play an important role in the synthesis of glycine and the conversion of this amino acid into another metabolite, serine [37]. Increased levels of glycine in CKD patients, especially those on stage 2, suggested that the loss of kidney function contributes to the accumulation of glycine in the circulation. Circulating concentrations of glycine have been found to be higher in people and rats with kidney dysfunction [38]. Lower levels of serine in CKD patients are also expected due to the association with a low conversion rate. Summers et al. [39] observed a reduction in serine in the plasma of CKD cats when compared to healthy animals. This corroborates with Brunetto et al. [40], who found a reduction in serine circulation in dogs with CKD stages 3 and 4 in a comparison with a control group. In the present study, we confirmed the association between increased levels of glycine in patients with reduced kidney function.

Another serum metabolite that showed accumulation in T60 of CKD2 patients when compared to the other two groups was fructose. The mechanism supporting this result may be attributed to the fact that this metabolite can be generated in the kidney during glucose reabsorption, as well as from intra-renal hypoxia that occurs in CKD [41,42]. Even though fructose may provide renal protection in cases of ischemia or high osmolarity, for example, high levels of this metabolite can induce intrarenal inflammation and fibrosis [41]. In addition, high serum fructose in human patients with CKD was associated with abnormalities in the TCA cycle [42].

The kidneys have a vital role in homeostasis, metabolism, and regulation of the serum concentration of amino acids. Glutamic acid is one of them, and it is correlated with kidney production as well as the accumulation as a result of decreased renal uptake [43,44,45]. In human medicine it was increased in patients with normal serum creatinine, but with some early impairment of renal function. The authors suggested that this metabolite may be utilized as a future biomarker for early stages of CKD [44]. We observed a strong correlation between glutamic acid accumulation and the second stage of CKD in this study. However, cats on stage 1 showed no difference compared to other groups, probably because the initial stage in this species did not show loss of renal function that compromises the uptake of this amino acid.

Arachidonic acid is a major component of cell membrane lipids, and is mainly metabolized by three enzymes: cyclooxygenase (COX), lipoxygenase (LOX), and cytochrome P450 (CYP450) [46]. Based on these metabolic pathways, this metabolite is a precursor of several pro-inflammatory mediators. When cells are under stress, arachidonic acid is released from phospholipids by phospholipase enzymes [46,47]. Hence, arachidonic acid metabolism and kidney inflammation are correlated in several ways [46,48]. Similar results have been found in the present study, as the CKD2 group showed a higher concentration of this metabolite.

Stearic acid is linked to the fact that, in CKD patients, the fatty acids oxidation is decreased [35]. This metabolite also showed a negative correlation when associated with a glomerular filtration rate (GFR), with high serum accumulation in the CKD2 group when compared to healthy animals. In humans and mouse models with tubulointerstitial fibrosis, Kang et al. [49] found that they had a lower expression of key enzymes and regulators of fatty acid oxidation (FAO) and a higher intracellular lipid deposition, including stearic acid, when compared to controls.

Creatinine is the most common metabolite studied in nephology, and as expected, higher creatinine was also observed in CKD2 patients when compared to CKD1 and control groups. It is an amino acid from muscular catabolism, derived from the metabolism of phosphocreatine, freely filtered by the glomerulus, and secreted to a small degree by proximal tubular cells, being a more specific biomarker used to estimate renal function due to the association with GFR [50,51,52,53]. Creatinine production is directly related to the body muscle mass and can also be influenced by dietary intake [54], variation in tubular secretion, and extrarenal creatinine excretion [50,55,56].

Increases in urea levels in CKD2 cats were also expected in this study. However, 60 days after the renal diet, urea had a higher concentration in the control group when compared to CKD2. This result may be linked to the composition of the diet used in this study and its metabolism according to each group, since all animals received the same diet at the same time. Previous studies with CKD cats have shown that blood urea decreased when they were feed with fermentable fiber from fructooligosaccharides when compared to the control cats, mainly because the health status of the cats influences the effects of fermentable fibers in the plasma metabolome and in the fecal microbiome [57]. The inclusion of galactooligosaccharides and beta-glucans on the renal diet suggested that these prebiotics may have influenced the urea metabolism due to the possible modulation of the microbiota, as mentioned before. Although microbiota analysis is necessary to affirm that, it is possible that the growth of bacteria that utilize ammonia from protein metabolism in the intestine may have resulted in lower intestinal absorption of this metabolite, which in turn reduced urea formation in the liver of CKD2 cats [58,59,60].

The limitations of the present study include a reduced sample size and the lack of a negative control group for comparison. 

## 5. Conclusions

Healthy and CKD cats 1 and 2 showed differences in the serum metabolomics profile when evaluated at time zero and after 60 days of the renal diet. Metabolomics markers have the potential use as possible early diagnostic biomarkers of chronic kidney disease, identifying early stages of CKD, and providing insights into the possible pathophysiologic processes that contribute to the development and progression of CKD.

## Figures and Tables

**Table 1:** Diet composition per 100 kcal of dry matter (DM) and ingredients ^1^ according to the manufacturer.

Nutrients (g/100 kcal DM)	Senior Diet	Renal Test Diet
Protein	10.34	8.68
Fat	5.23	3.94
Crude fiber	0.62	0.41
Ash	1.87	1.08
Calcium	0.32	0.13
Phosphorus	0.29	0.12
Ca/P ratio	1.12	1.08
Potassium	0.17	0.22
Sodium	0.20	0.08
Omega-3	0.10	0.28
Metabolizable energy (kcal/kg)	3.920	4.353
Essential amino acids		
Arginine	0.70	0.48
Phenylalanine	0.49	0.36
Histidine	0.26	0.18
Isoleucine	0.42	0.37
Leucine	0.94	0.79
Lysine	0.59	0.49
Methionine	0.28	0.17
Taurine	0.07	0.05
Threonine	0.41	0.35
Tryptophan	0.08	0.09
Valine	0.52	0.46

^1^ Ingredients: Senior diet: poultry meal, pork protein isolate, corn gluten meal, egg product, broken rice, beet pulp, oat groats, chicken fat, soy oil, fish oil, hydrolyzed poultry and pork, sodium chloride, potassium chloride, antioxidants butylated hydroxyanisole and butylated hydroxytoluene, betaine, L-carnitine, L-lysine, fructooligosaccharide, mannooligosaccharide, sugarcane fiber, acidifying additive, chondroitin sulfate, glucosamine sulfate, taurine, Yucca schidigera extract, dried brewer’s yeast, vitamin and mineral premix; Renal test diet: albumine, hydrolyzed chicken meal, poultry meal, pork protein isolate, corn gluten meal, egg product, soy protein isolate, barley, cassava flour, ground whole corn, soy lecithin, broken rice, beet pulp, poultry fat, pork fat, fish oil, calcium carbonate, potassium chloride, potassium citrate, fructooligosaccharide, galactooligosaccharide, mannooligosaccharide, sugarcane fiber, acidifying additive, antioxidants butylated hydroxyanisole and butylated hydroxytoluene, DL-methionine, L-lysine, magnesium oxide, calcium sulfate, vitamin and mineral premix.

**Table 2:** Description of age, body weight and biochemical serum profile at T0 and T60.

Variables	Control Group (*n* = 10)		CKD1 Group (*n* = 6)		CKD2 Group (*n* = 9)	
	T0	T60	T0	T60	T0	T60
Age (years)	5.30 ± 1.07	-	10.83 ± 1.05	-	10.22 ± 1.35	-
Body weight (kg)	4.52 ± 0.65	4.53 ± 0.58	5.29 ± 0.73	5.28 ± 0.67	4.72 ± 1.51	4.60 ± 1.58
Total protein (g/dL)	7.90 ± 0.57	7.68 ± 0.82	8.10 ± 0.60	7.87 ± 0.75	8.14 ± 0.42	7.92 ± 0.43
Albumin (g/dL)	3.30 ± 0.19	3.29 ± 0.41	3.60 ± 0.24	3.53 ± 0.14	3.50 ± 0.21	3.51 ± 0.28
Glucose (mg/dL)	79.40 ± 9.74	109 ± 34.98	81.67 ± 13.41	83 ± 6.87	79.67 ± 5.29	95.22 ± 37.65
Creatinine (mg/dL)	1.35 ± 0.21	1.16 ± 0.24	1.32 ± 0.12	1.29 ± 0.10	2.03 ± 0.32	1.94 ± 0.81
BUN (mg/dL)	24.67 ± 2.98	23.08 ± 2.43	23.42 ± 1.53	23.02 ± 2.08	35.06 ± 5.28	35.81 ± 11.81
SDMA (µg/dL)	9.56 ± 4.10	9.5 ± 3.37	10.60 ± 3.36	7.50 ± 1.05	14.44 ± 3.88	11.33 ± 5.94
Phosphorus (mg/dL)	5.58 ± 0.65	5.97 ± 0.84	4.83 ± 0.51	4.97 ± 0.57	5.08 ± 0.48	5.96 ± 1.01
Total calcium (mg/dL)	9.96 ± 0.51	9.43 ± 0.85	10.40 ± 0.32	10.22 ± 0.50	10.49 ± 0.31	10.02 ± 0.56
Sodium (mEq/L)	152 ± 2.11	156.40 ± 1.17	154 ± 2.53	156.17 ± 2.14	152.67 ± 2.00	157.33 ± 3.39
Potassium (mEq/L)	4.84 ± 0.31	5.01 ± 0.48	5.15 ± 0.57	4.68 ± 0.49	4.88 ± 0.50	5.13 ± 0.57
Chloride (mEq/L)	115.30 ± 3.74	120.20 ± 2.90	118 ± 2.28	121.33 ± 1.37	117.67 ± 1.32	122.67 ± 3.67
Cholesterol (mg/dL)	153.60 ± 44.82	129.20 ± 45.16	221.83 ± 40.48	200 ± 46.38	206.33 ± 58.19	163.56 ± 51.10
Tryglicerides (mg/dL)	46.50 ± 22.42	47 ± 15.96	61.50 ± 31.16	60.83 ± 12.12	52.78 ± 13.98	68.67 ± 36.53
ALP (mg/dL)	29.90 ± 7.00	26.50 ± 5.10	33.67 ± 8.04	32 ± 8.76	38.33 ± 24.35	37.33 ± 27.80
ALT (mg/dL)	65.60 ± 20.32	56.80 ± 18.96	65.17 ± 19.34	57 ± 14.39	82.67 ± 61.80	76.56 ± 50.94

BUN, blood urea nitrogen; SDMA, symmetric dimethylarginine; ALP, alkaline phosphatase; ALT, alanine aminotransferase.

**Table 3:** Description of all metabolites identified in the serum, grouped by their biochemical classes.

Biochemical Class	Compound Names	Code	Identification Level	Identification in the Human Metabolome Database (HMDB)	Relative Standard Deviation (%)
Amino acids and derivatives	L-Alanine	ALA	2	0000161	7.3
	L-Valine	VAL	2	0000883	7.1
	L-Isoleucine	ILE	2	0000172	7.0
	L-Proline	PRO	2	0000162	11.0
	L-Glycine	GLY	2	0000123	10.1
	L-Serine	SER	2	0000187	14.2
	L-Threonine	THR	2	0000167	4.8
	L-Homoserine	hSER	2	0000719	23.3
	5-Oxoproline	PYR	2	0000267	19.7
	Creatinine	CRE	2	0000562	23.2
	L-Glutamic acid	GLU	2	0000148	6.5
	L-Phenylalanine	PHE	2	0000159	10.1
	L-Arginine	ARG	2	0000517	9.8
	L-Tyrosine	TYR	2	0000158	22.7
	L-Tryptophan	TRP	2	0000929	25.3
Carbohydrates and conjugates	D-Fructose	FRU	2	0000660	25.3
	D-Ribose	RIB	2	0000283	16.2
	D-Mannose	MAN	2	000169	10.5
	D-Allose	ALLO	2	0001151	10.5
	Inositol-Phosphate	INO	2	0000213	23.4
	Lactose	LAC	2	0000186	27.3
Fatty acids	Palmitoleic acid	C16:1	2	0003229	24.5
	Palmitic acid	C16:0	2	0000220	11.9
	Heptadecanoic acid	HEP	2	0002259	18.3
	Linoleic acid	LNL	2	0000673	25.3
	Oleic acid	OLC	2	0000207	19.4
	Stearic acic	STE	2	0000827	13.5
	Arachidonic acid	ARA	2	0001043	15.0
	Docosahexaenoic acid	DHA	2	0002183	7.0
	Arachidic acid	C20:0	2	0002212	22.1
	1-Monopalmitin	MG16:0	2	0011564	13.6
	Monostearin	MG18:0	2	0011131	15.3
Prenol Lipids	Alpha-Tocopherol	αTOC	2	0001893	14.7
Steroids	Cholesterol	CHO	2	0000067	13.5
Carboxilic acid	Citric acid	CIT	2	0000094	29.4
	Sarcosine	SAR	2	0000271	21.3
Hydroxy acids	3-Hydroxybutyric acid	3HBT	2	0000011	11.0
Organic Carbonic acids	Urea	URE	2	0000294	15.8
Imidazopyrimidines	Uric acid	URI	2	0000289	24.3
Purine nucleosides	Guanosine	GUA	2	0000133	18.1
Indoles	Serotonin	5HTA	2	0000259	12.6
Glycerophospholipids	Glycerol-3-Phosphate	G3P	2	0000126	22.5
Benzene	3-Hydroxyanthranilic acid	3OHAA	2	0001476	28.9

**Table 4:** Results obtained by univariate analysis of serum metabolites at T0 and T60.

Metabolites	*p* Value	FDR	Tukey’s HSD
Citric acid	0.0010	0.0440	CKD II T0-CKD I T0; Control T0-CKD II T0
Monostearin	0.0021	0.0444	Control T0-CKD II T0
Glycine	0.0003	0.0055	Control T60-CKD I T60; Control T60-CKD II T60
Fructose	0.0007	0.0107	CKD II T60-CKD I T60; Control T60-CKD II T60
Glutamic acid	0.0013	0.0144	Control T60-CKD II T60
Arachidonic acid	0.0042	0.0269	Control T60-CKD II T60
Stearic acid	0.0044	0.0269	Control T60-CKD II T60
Creatinine	0.0358	0.0002	CKD II T60-CKD I T60; Control T60-CKD II T60
Urea	0.0031	0.0265	Control T60-CKD II T60

FDR = False Discovery Rate; HDS = Tukey’s Honest Significant Difference Test; CKD I T0 = CKD cats stage 1 before being fed a renal diet; CKD II T0 = CKD cats stage 2 before being fed a renal diet; Control T0 = control group (healthy animals) before being fed a renal diet; CKD I T60 = CKD cats stage 1 after being fed a renal diet; CKD II T60 = CKD cats stage 2 after being fed a renal diet; Control T60 = control group (healthy animals) after being fed a renal diet.

## Data Availability

The data presented in this study are available on request from the corresponding author. The data are not publicly available due to restrictions de privacy and ethical.
